# Role of TFEB Mediated Autophagy, Oxidative Stress, Inflammation, and Cell Death in Endotoxin Induced Myocardial Toxicity of Young and Aged Mice

**DOI:** 10.1155/2016/5380319

**Published:** 2016-04-21

**Authors:** Fang Li, Fangfang Lang, Huilin Zhang, Liangdong Xu, Yidan Wang, Enkui Hao

**Affiliations:** ^1^Department of Health, Jinan Central Hospital, Shandong University, Jinan, China; ^2^Department of Obstetrics and Gynecology, Jinan Central Hospital, Shandong University, Jinan, China; ^3^Central Laboratory, Jinan Central Hospital, Shandong University, Jinan, China; ^4^Department of Cardiology, Qianfoshan Hospital, Shandong University, Jinan, China

## Abstract

Elderly patients are susceptible to sepsis. LPS induced myocardial injury is a widely used animal model to assess sepsis induced cardiac dysfunction. The age dependent mechanisms behind sepsis susceptibility were not studied. We analyzed age associated changes to cardiac function, cell death, inflammation, oxidative stress, and autophagy in LPS induced myocardial injury. Both young and aged C57BL/6 mice were used for LPS administration. The results demonstrated that LPS induced more cardiac injury (creatine kinase, lactate dehydrogenase, troponin I, and cardiac myosin-light chains 1), cardiac dysfunction (left ventricular inner dimension, LVID, and ejection fraction (EF)), cell death, inflammation, and oxidative stress in aged mice compared to young mice. However, a significant age dependent decline in autophagy was observed. Translocation of Transcription Factor EB (TFEB) to nucleus and formation of LC3-II were significantly reduced in LPS administered aged mice compared to young ones. In addition to that, downstream effector of TFEB, LAMP-1, was induced in response to LPS challenge in young mice. The present study newly demonstrates that TFEB mediated autophagy is crucial for protection against LPS induced myocardial injury particularly in aging senescent heart. Targeting this autophagy-oxidative stress-inflammation-cell death axis may provide a novel therapeutic strategy for cardioprotection in the elderly.

## 1. Introduction

Sepsis is a leading cause of death among critically ill patients and elderly patients are most vulnerable to it [1, 2]. The elderly population will grow more rapidly and the world's elderly population will cross that of the young by 2050 when sepsis in those patients will be priority [3, 4]. Heart failure is a well-known complication of sepsis and also known as septic cardiomyopathy. The mechanism of septic cardiomyopathy has been studied well and a series of molecular mechanisms such as apoptosis, cytokines, immune regulation, toxin, mitochondria, and energy metabolism has been implicated. However, the precise mechanisms and their role in the pathogenesis of septic cardiomyopathy in aging remain incompletely understood.

Autophagy is an intracellular process of protein degradation and recycling. Autophagic deregulation leads to many diseases (neurodegenerative disorder, cancer, etc.) and protects against oxidative damage and inflammation [5]. Reduced autophagic potential leads to aging and increased autophagy delays aging [6, 7]. Autophagy is crucial to maintaining homeostasis in the heart, and a decline is associated with accelerated cardiac aging [8]. However, the role of autophagy in sepsis and associated cardiac dysfunction are not clearly understandable till date.

Transcription factor EB (TFEB) is one of the regulators of autophagy. TFEB translocates to nucleus and regulates hundreds of genes which consist of Coordinated Lysosomal Expression and Regulation (CLEAR) network [9, 10]. Those CLEAR networks genes are involved in autophagosomes formation (such as LAMP-1, VPS11), vesicle formation and elongation (such as MAP1LC3), and cargo recognition and degradation (such as SQSTM1 or p62).

Cardiac aging leads to structural, functional changes in addition to cellular and molecular changes [11]. Oxidative stress is key contributor of cardiovascular aging at the molecular level [11]. In aging heart, the majority of ROS are derived from NOX (NADPH oxidase) and mitochondrial electron transport chain [12]. There is a close link between oxidation and inflammation and, as aging occurs, more oxidative/nitrative damaged biomolecules accumulated in the heart, which lead to more inflammation [13].

Here, we examined the mechanism of sepsis associated cardiac dysfunction in aging and autophagy-oxidative stress-inflammation axis played critical role in LPS induced cardiac dysfunction in aged mice.

## 2. Methods

### 2.1. Animal Treatments

Male C57BL/6 mice that are 4–6-week-old (young) and 22–24-week-old (aged) were obtained from the Experimental Animal Center of Shandong University (Jinan, Shandong, China). LPS was purchased from Sigma in China (Beijing, China). LPS was dissolved in normal saline and administered intraperitoneally (i.p.) at a volume of 10 *μ*L/gram for each mouse. The mice were given 4 mg/kg of LPS for 18 hours.

Mice experimental protocols were approved by Institutional Animal Care and Use Committee of Shandong University and were in compliance with Health Ministry of the People's Republic of China. Mice were sacrificed under isoflurane (5%) deep anesthesia after completion of echocardiography.

### 2.2. Echocardiography

Mice were anesthetized with isoflurane (1%) mixed with oxygen. Echocardiographic cardiac parameters were determined by VisualSonics Vevo770 system (VisualSonics, Inc., Toronto, Canada) as described earlier [14, 15].

### 2.3. Real-Time PCR

mRNA level of TNF*α* (tumor necrosis factor), IL1*β* (interleukin 1 beta), MIP1*α* (macrophage inflammatory protein-1 alpha), MCP1 (CD46), MAP1lc3 (microtubule-associated protein 1 light chain 3), VPS11 (vacuolar protein sorting-associated protein 11), or *β* actin was detected by reverse transcription and real-time PCR. Total RNA was isolated by Trizol method as described earlier [8]. All predesigned primers were purchased from Qiagen. The fold changes were calculated based on relative quantification method [16].

### 2.4. Western Blot

Heart tissues were homogenized in lysis buffer and protein concentration was determined as described earlier [14].

Western transfer in PVDF membrane was performed after running equal amount of proteins in SDS-PAGE. Membranes were probed with LC3 antibody, GAPDH antibody (1 : 200, Cell Signaling Technology), anti-TFEB antibody (1 : 200, Santa Cruz Biotechnology), anti-LAMP1 antibody (1 : 200, Sigma-Aldrich), and Histone H3 (1 : 200, Santa Cruz Biotechnology) overnight at 4°C. The membranes were probed with HRP-conjugated secondary antibody (1 : 2000, Rockland, Gilbertsville) for 1 h at room temperature. The chemiluminescence in the membranes was analyzed on X-ray film.

### 2.5. Immunohistochemistry

Histological analyses were performed on paraffin embedded section. After deparaffinization and antigen retrieval process, sections were stained with anti-nitrotyrosine antibody overnight and developed with VECTASTAIN Elite ABC Kit Rabbit IgG and ImmPACT DAB Peroxidase (HRP) Substrate (Vector Laboratories) according to manufacturer's instruction.

### 2.6. DNA Fragmentation

DNA fragmentation was measured by ELISA based kit (Roche) according to manufacturer's instruction.

### 2.7. PARP Activity

PARP activity, we used the HT Universal Colorimetric PARP assay kit from Trevigen as described earlier [17].

### 2.8. Protein Nitrotyrosine Nitration

Protein nitrotyrosine nitration was determined using OxiSelect*™* Nitrotyrosine ELISA Kit (Cell Biolabs) according to manufacturer's instruction.

### 2.9. Protein Carbonyl Content

Carbonyl content in protein from tissue lysate was determined by Protein Carbonyl Colorimetric Assay Kit (Cayman Chemical) according to manufacturer's recommendation.

### 2.10. Statistical Analysis

Data were expressed as mean ± standard deviation (SD), and statistical analysis was done by using GraphPad Prism software. Paired *t*-test or one-way analysis of variance followed by Tukey's Post Test were performed. *P* < 0.05 was considered statistically significant.

## 3. Results and Discussion

### 3.1. Aged Mice Are Prone to LPS Induced Cardiac Dysfunction

**Figure 1:**
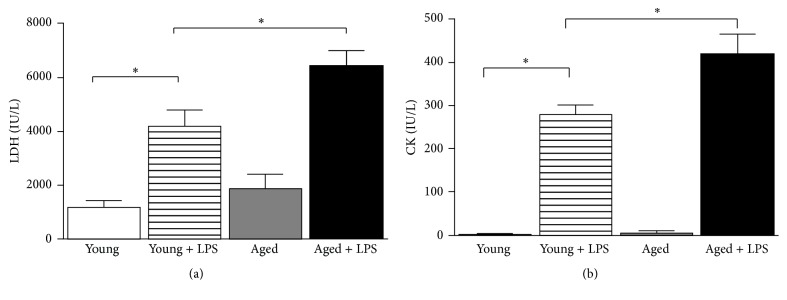
Effect of LPS in young and aged mice on cardiac injury. Cardiac injury was measured by plasma LDH (a) and CK (b). Both enzymes were significantly increased in LPS treated mice. Increase of LPS induced cardiac injury in aged mice was significantly higher than young ones. Values represented as means ± SD; ^*∗*^ *P* < 0.05 and *n* = 6/group.

**Figure 2:**
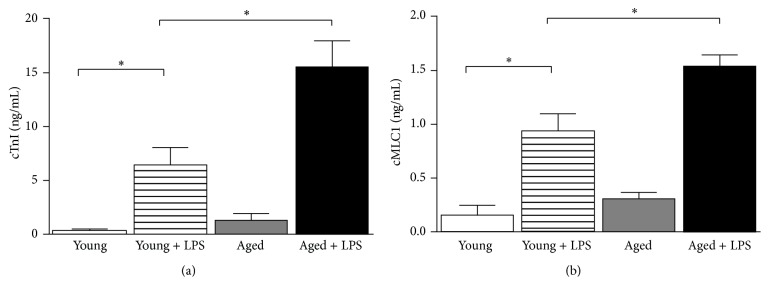
Effect of LPS in young and aged mice on cardiac damage. Cardiac damage was measured by plasma cTnI (a) and cMLC1 (b), which were secreted by damaged heart. Both markers were significantly increased in LPS treated mice. Increase of LPS induced cardiac damage in aged mice was significantly higher than young ones. Values represented as means ± SD; ^*∗*^ *P* < 0.05 and *n* = 6/group.

To examine whether young mice confer more cardiac protective effects in vivo than aged mice, we used both young and aged C57BL/6 mice. LPS was administered intraperitoneally at 4 mg/kg overnight for about 18 hours, which caused marked myocardial tissue damage in young and aged mice as evidenced by elevated plasma lactate dehydrogenase (LDH) and creatine kinase (CK) in Figure 1. The tissue damage was more significant in aged mice compared to young mice. Cardiac troponin I (cTnI) and cardiac myosin-light chains 1 (cMLC1) testing are an essential component of acute heart disorders in particular as a highly specific marker for myocardial infarction or heart muscle cell death. In young group of mice cTnI level was increased from 0.36 to 6.46 (ng/mL) whereas in aged mice plasma of cTnI was increased from 1.29 to the level of 15.5 (ng/mL) when LPS was administered (Figure 2(a)). Similarly, in young group of mice, cMLC1 level was increased from 0.15 to 0.92 (ng/mL) whereas in aged mice plasma of the same was increased from 0.30 to 1.54 (ng/mL) (Figure 2(b)). Similar pattern was observed earlier in other cardiac injury models [18].

**Figure 3:**
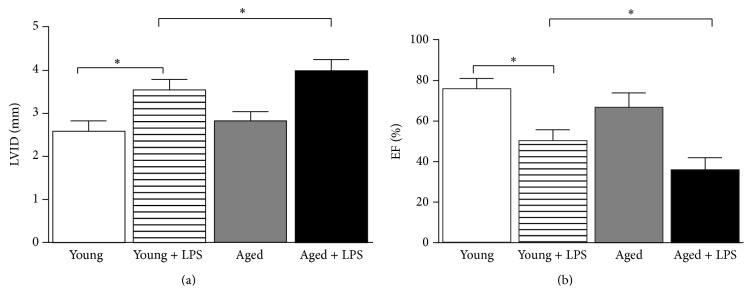
Effect of LPS in young and aged mice on cardiac function. Cardiac function parameters left ventricular internal dimension (LVID, (a)) and ejection fraction (EF, (b)) were measured by echocardiography. LVID was significantly increased whereas EF was decreased in LPS treated mice. The difference of LPS induced cardiac dysfunction in aged mice was significantly higher than young ones. Values represented as means ± SD; ^*∗*^ *P* < 0.05 and *n* = 6/group.

Left ventricular (LV) structure and function were assessed by echocardiography. As shown in Figures 3(a) and 3(b), LPS caused an increase in end-diastolic left ventricular inner dimension (LVID) and a decrease in ejection fraction (EF) in both young and old mice. However cardiac dysfunction was more significant in older mice compared to young mice.

We have demonstrated earlier that LPS induced significant cardiac dysfunction [14]. The structural and functional alterations in aging hearts are indications of failing heart, which thus may increase the vulnerability of the aging heart to develop heart failure [19, 20]. In patients with septic shock dilation of left ventricles is reported [21]. Other hemodynamic profile of septic shock includes elevated cardiac index and reduced systemic vascular resistance [22]. In this study, we observed that aging heart was more susceptible to LPS induced myocardial toxicity. Notably cardiac diastolic dysfunction is associated with aging [23, 24]. We demonstrated that two key parameters, LVID and EF, of diastolic function were modulated with aging and significantly altered in LPS induced myocardial toxicity. It is reported in studies with elderly and younger individuals of similar physical status that the end-diastolic volume and ejection fraction are increased during exercise [25, 26]. Major components of cardiovascular aging are decrease in elasticity and an increase in stiffness of the arterial system that leads to systolic blood pressure and left ventricular hypertrophy and alteration in the left ventricular wall [27]. However, in mice model, we observed difference in diastolic function, which was altered by endotoxin.

### 3.2. Cardiac Cell Death Increases with Aging in LPS Induced Cardiac Dysfunction

**Figure 4:**
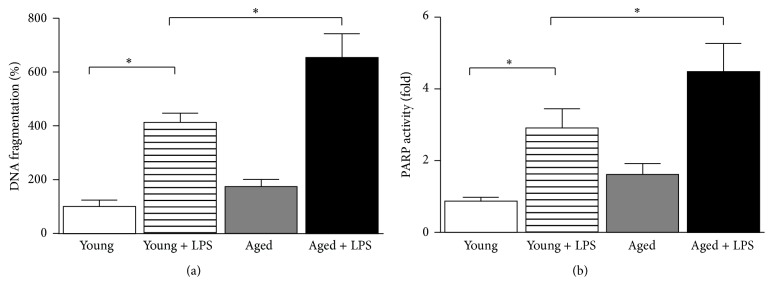
Effect of LPS in young and aged mice on cardiac cell death. Cardiac cell death markers DNA fragmentation (a) by quantitative ELISA and PARP activity assay (b). Both markers were significantly increased in LPS treated mice. Increase of LPS induced cardiac damage in aged mice was significantly higher than young ones. Values represented as means ± SD; ^*∗*^ *P* < 0.05 and *n* = 6/group.

As we and others have shown earlier that cardiac cell death leads to cardiac dysfunction, we compared cell death between young and aged mice upon LPS administration [14]. LPS induced 412% and 654% increase of DNA fragmentation in young and aged mice, respectively (Figure 4). We also examine PARP activity, which also is marker for cell death. PARP activity was increased 2.9-fold in young mice upon LPS administration whereas it increased 4.5-fold in aged mice.

One of the key factors of cardiac dysfunction is cardiomyocytes cell death [28]. Two types of cell death (apoptosis and regulated necrosis) were associated with cardiac dysfunction [28]. We used two distinctive markers such as DNA fragmentation and PARP activity as representation of these types of cell death. Cardiac dysfunction was correlated with cell death data in both young and aged mice. The endotoxin induced cardiomyocytes cell death is primarily apoptotic in nature [14]. However, the process of endotoxin induced cell death is much more complex involving apoptosis, necrosis, pyroptosis, and oncosis [29]. The level of endotoxin and its timing play critical role in determining the prevalence of one pathway over the other.

### 3.3. Increase of Inflammatory Pathway with Aging in LPS Induced Cardiac Dysfunction

**Figure 5:**
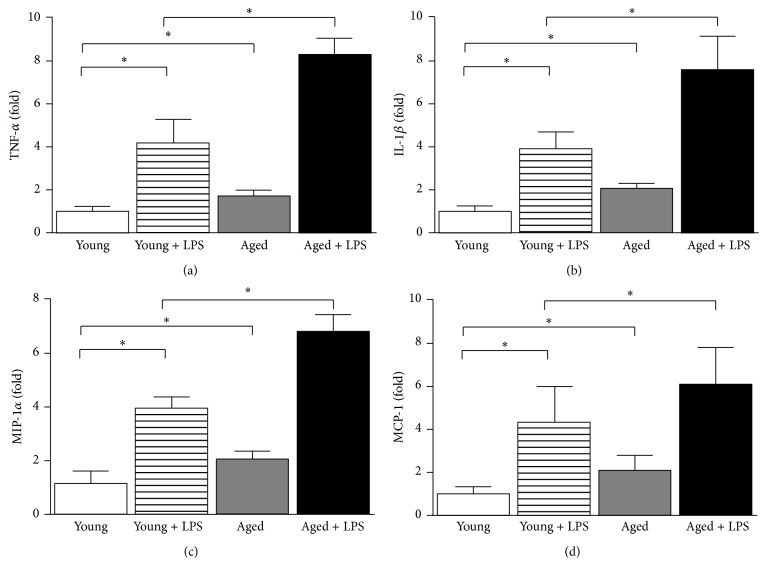
Effect of LPS in young and aged mice on cardiac inflammation. Cardiac inflammation markers TNF-*α* (a), IL-1*β* (b), MIP-1*α* (c), and MCP (d) were measured by real-time PCR. All markers were significantly increased in LPS treated mice. Increase of LPS induced cardiac inflammation in aged mice was significantly higher than young ones. Values represented as means ± SD; ^*∗*^ *P* < 0.05 and *n* = 6/group.

The expression of four proinflammatory cytokines in heart was examined by real-time PCR. As shown in Figure 5, LPS induced all four cytokines in both young and aged mice. The effect of LPS on aged mice was more significant than younger mice. We also observed that basal expression of inflammatory cytokines in aged heart is higher compared to young heart. TNF*α* mRNA increased to 4.2-, 1.7-, and 8.3-fold in the hearts of young with LPS, aged, and aged with LPS group, respectively (Figure 5(a)). IL-1*β* mRNA increased to 3.9-, 2.0- and 7.6-fold in the hearts of young with LPS, aged, and aged with LPS group, respectively (Figure 5(b)). MIP-1*α* mRNA increased to 3.8-, 2.1-, and 6.8-fold in the hearts of young with LPS, aged, and aged with LPS group, respectively (Figure 5(c)). MCP mRNA increased to 4.34-, 2.09-, and 6.1-fold in the hearts of young with LPS, aged, and aged with LPS group, respectively (Figure 5(d)).

Inflammatory pathways are responsible for cell death associated cardiac dysfunction in aging heart [30, 31]. Our study also demonstrated that a significant increase of inflammation is correlated with cell death in aging heart. Aging association with chronic inflammation in heart has been reported earlier in addition to low level systemic inflammation [11]. Our data is consistent with earlier publications. In cardiovascular aging, inflammation is associated with other cardiovascular disease and induced by many stimulus [32]. One of such key pathways is TNF*α* signaling and associated NF-*κ*B (nuclear factor kappa-B) activation [33]. Inflammation contributes to pathogenesis in a range of cardiac conditions but interventional approach with anti-inflammatory is not promising [32]. Excessive inflammation is a major cause of heart failure in sepsis and mitochondria play significant role [34]. In response to endotoxin, mitochondria produce large bust of reactive oxygen species (ROS), which target protein, lipid, and DNA in the cell [14]. Heart is specifically prone to mitochondrial stress as mitochondria consist of one-third volume and age dependent mitochondrial damage is mainly caused by its ROS [34].

### 3.4. Increase of Oxidative/Nitrative Stress with Aging in LPS Induced Cardiac Dysfunction

**Figure 6:**
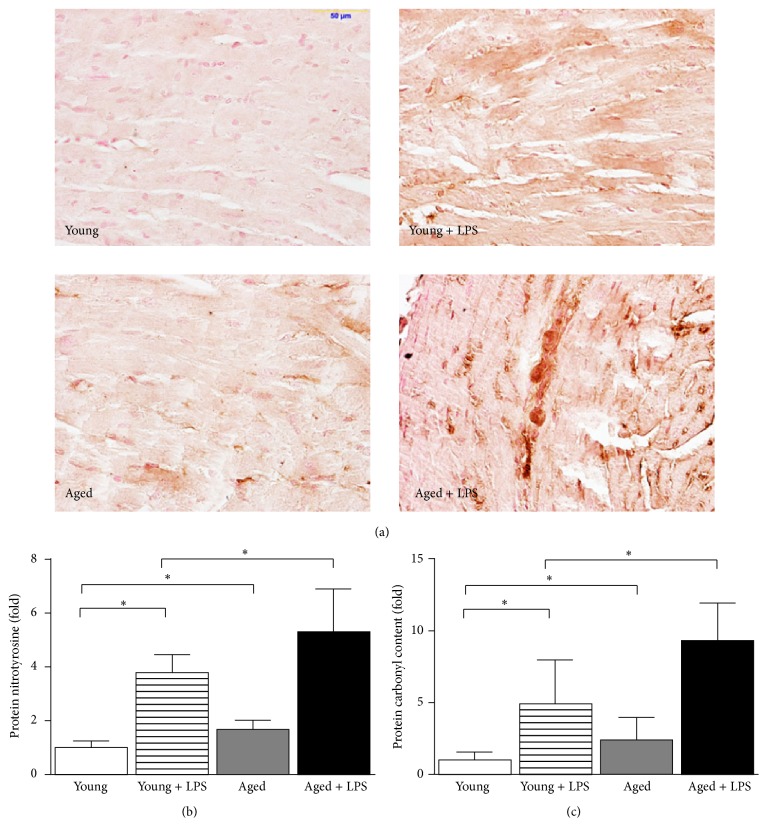
Effect of LPS in young and aged mice on cardiac oxidative damage. Cardiac oxidative markers protein nitration (a) by histology and protein nitrotyrosine (b) and carbonyl (c) content measured by quantitative ELISA. All markers were significantly increased in LPS treated mice. Increase of LPS induced cardiac inflammation in aged mice was significantly higher than young ones. Values represented as means ± SD; ^*∗*^ *P* < 0.05 and *n* = 6/group.

LPS administration led to increased reactive oxygen species (ROS) production in both young and aged mice as evidenced by protein nitration and carbonyl content. Protein nitration or protein nitrotyrosine nitration is well-known marker for ROS production [35]. Histological staining of paraffin embedded section of hearts demonstrated distinct pattern in both LPS treated young and aged mice (Figure 6(a)). The level of staining in aged mice is significantly stronger than young mice upon LPS administration. Quantitative determination of same protein nitrotyrosine marker by ELISA demonstrated that LPS induced more protein nitrotyrosine nitration in old mice compared to young mice and there is basal level increase of protein nitrotyrosine in aged mice.

The major contributors of oxidative/nitrative stress in heart are the reactive oxygen species (ROS) family of molecules including superoxide anion, lipid radicals, nitric oxide, hydrogen peroxide, and peroxynitrite [36]. The method of detection of ROS is technically difficult in live animals; therefore most studies were focused on oxidative footprints such as protein nitrotyrosine nitration and protein carbonyl modification [37]. Here, we demonstrated by two independent methods that LPS induced oxidative stress in heart and such effect was significantly enhanced in aging.

### 3.5. Inefficient Autophagy in Aging Correlates to Increase of LPS Induced Cardiac Cell Death

**Figure 7:**
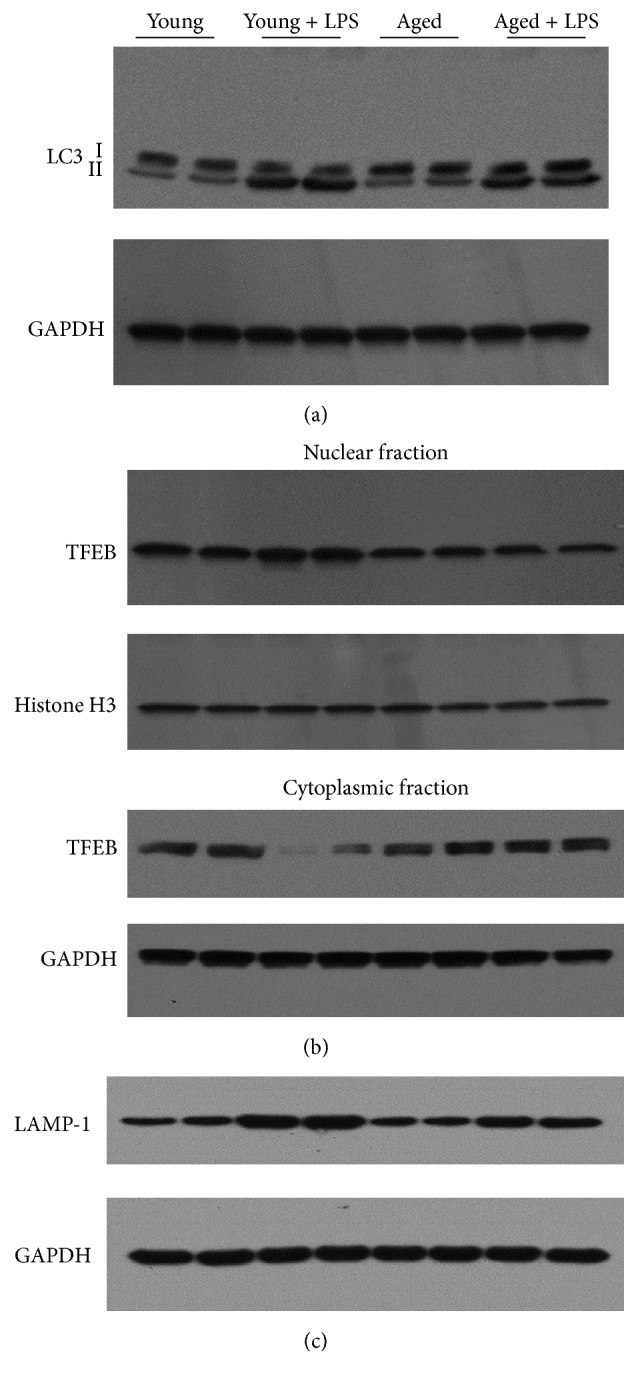
Effect of LPS in young and aged mice on cardiac autophagy. Markers of autophagy LC3 with loading control GAPDH were determined by western blot analyses (a). Both nuclear and cytoplasmic fractions of TFEB, along with nuclear specific marker Histone H3 and cytoplasmic marker GAPDH, were determined by western blot experiments (b). LAMP-1, downstream effector of TFEB and involved in lysosomal biogenesis, was also analyzed (c). Significant autophagy response in LPS treated mice was observed. However, LPS induced cardiac autophagic response in aged mice was significantly lower than young ones.

**Figure 8:**
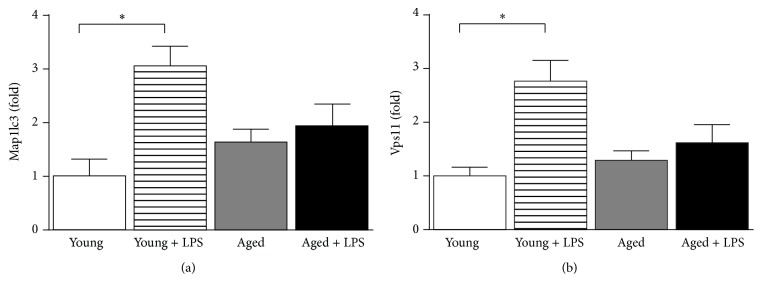
Effect of LPS in young and aged mice in downstream effector genes of TFEB. TFEB regulated genes Map1lc3 and Vps11 were examined at mRNA level by real-time PCR. LPS induced MRNA level in both genes in young mice whereas such pattern is absent in old mice. Values represented as means ± SD; ^*∗*^ *P* < 0.05 and *n* = 6/group.

Autophagy maintains cell homeostasis in heart under starvation, remodeling, and aging [38]. We have observed that LPS administration led to induction of autophagy (using LC3II marker) in both young and aged mice (Figure 7). However, level of LCII is much less in aged mice compared to young mice upon LPS administration. These results demonstrated that autophagy machinery might be inefficient in heart of aging mice compared to young ones. The transcription factor EB (TFEB), a regulator of autophagy and its nuclear localization, leads to positive regulation of CLEAR (Coordinated Lysosomal Expression and Regulation) network [39]. We also examined whether TFEB has any role in LPS induced myocardial toxicity particularly in aging. We found that LPS induced significant nuclear localization of TFEB in young mice whereas such increase is clearly absent in aged mice. In cytoplasmic fraction a corresponding decrease in TFEB content was observed in LPS treated young mice and such distinct pattern was absent in aged mice. We also examined the downstream effector of TFEB and key protein in lysosomal biogenesis, LAMP-1, and it was induced significantly in young mice when administered with LPS (Figure 7(c)). However, there was little increase in LAMP-1 in aged mice when administered with LPS and the changes were lower than that of young ones. We further analyzed Map1lc3 and Vps11, well-known targets of TFEB, by real-time PCR (Figure 8). LPS induced those gene expressions in young mice whereas such induction was absent in old mice.

Oxidative stress in cardiomyocytes has been reported to serve as important stimuli of autophagy in response to cell stress [40]. LPS induced oxidative stress might lead to autophagy. Autophagy is a protection and remodeling mechanism to protect cardiomyocytes against cell stress [41]. We have observed that autophagy is significantly impaired in aged mice with LPS compared to young ones whereas cardiomyocytes cell death and cardiac dysfunction are highly pronounced in aging heart with LPS. Our data suggested that inefficient autophagy machinery in aging is a key determining factor in protection against LPS induced myocardial toxicity. Autophagy also plays cardioprotective role in other cardiac injury models [42, 43]. To understand further the autophagic machinery, we examined the role of TFEB in the process. TFEB is master regulator of CLEAR gene network consisting of over 400 genes, which encoded proteins for lysosomal biogenesis and function. These are important part of autophagic machinery [10]. We also observed TFEB mediated regulation of LAMP-1 protein and gene expression of Map1lc3 and Vps11. All these markers are also key members of CLEAR network. In response to cellular stress, TFEB translocates to nucleus and activates lysosomal biogenesis genes [44, 45]. Here we observed that, despite cellular stress with LPS, TFEB did not translocate to nucleus in aged mice, thus preventing the network for switching to autophagy machinery.

Autophagy is important for repair of cellular and also involved in self-killing of irreversibly injured cells. Programmed cell death is also classified into PCD1 (apoptosis) and PCD2 (autophagic cell death) [46]. Both pathways have overlapping mechanisms and are involved in cardiomyocytes cell death [47].

**Figure 9:**
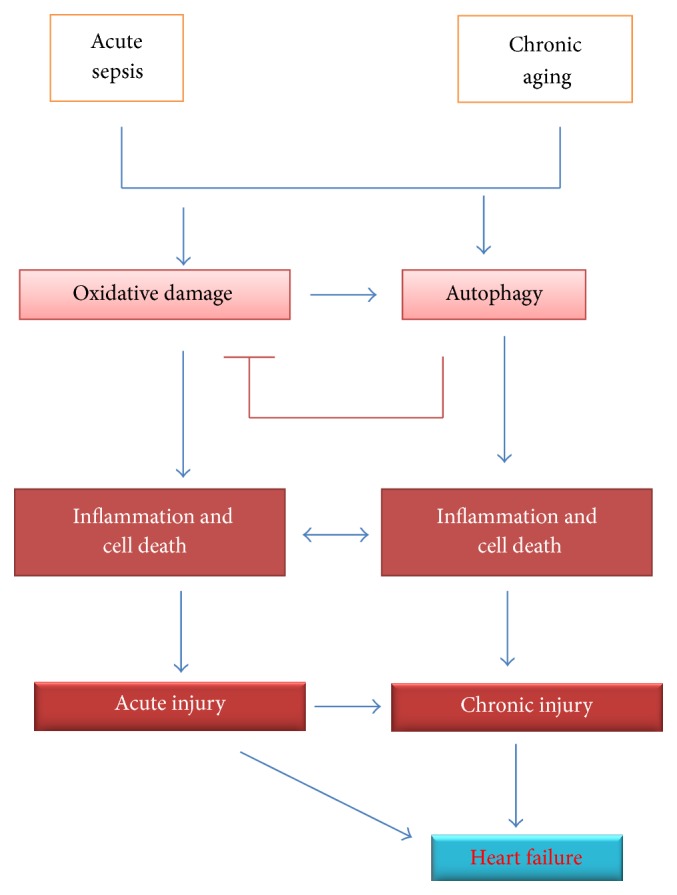
Schematic diagram of LPS induced heart failure in young and aged mice. Aging led to oxidative damage which may induce autophagy, where autophagic response leads to less oxidative damage. Oxidative damage leads to inflammation and cell death and is more pronounced in aging. This leads to chronic injury and associated cardiac dysfunction or heart failure.

Autophagy leads to cardioprotection by removing misfolded protein or dysfunctional mitochondria or maintaining energy homeostasis [41]. In sepsis, a burst of ROS leads to oxidative damage which leads to inflammation and cell death followed by acute cardiac injury. Oxidative damage also leads to induction of autophagy machinery and autophagy leads to protection against oxidative damage by removing damaged mitochondria (Figure 9). In aging, a higher basal level of oxidative stress and inflammation exists and LPS further exacerbated those stimuli which lead to profound cell death, chronic injury, and associated cardiac dysfunction (Figure 9).

## 4. Conclusion

We demonstrated for the first time that TFEB mediated autophagy played critical role in age dependent LPS induced myocardial toxicity which led to cardiac dysfunction. In aging heart, the translocation of TFEB to nucleus was greatly impaired and the autophagy machinery was impaired. Due to impaired autophagy, the basal level of inflammation and oxidative stress is higher in aging heart, which was further escalated in response to LPS.
